# Effects of 3-week repeated cold water immersion on leukocyte counts and cardiovascular factors: an exploratory study

**DOI:** 10.3389/fphys.2023.1197585

**Published:** 2023-08-29

**Authors:** Ninja Versteeg, Ron Clijsen, Erich Hohenauer

**Affiliations:** ^1^ Rehabilitation and Exercise Science Laboratory (RESlab), Department of Business Economics, Health and Social Care, University of Applied Sciences and Arts of Southern Switzerland, Landquart, Switzerland; ^2^ International University of Applied Sciences THIM, Landquart, Switzerland; ^3^ Department of Movement and Sport Sciences, Vrije Universiteit Brussel, Brussels, Belgium; ^4^ Department of Health, Bern University of Applied Sciences, Berne, Switzerland; ^5^ Department of Neurosciences and Movement Science, University of Fribourg, Fribourg, Switzerland

**Keywords:** cold water immersion, cryotherapy, immune system, blood count, leukocytes, cardiovascular factors

## Abstract

**Aim:** This exploratory study aimed to investigate the effects of a 3-week repeated cold water immersion (CWI) intervention on leukocyte counts and cardiovascular factors (mean arterial pressure [MAP], heart rate [HR]) in healthy men.

**Methods:** A total of *n* = 12, non-cold-adapted men (age: 25.2 ± 4.0 years; height: 177.8 ± 5.6 cm; weight: 73.8 ± 6.5 kg) were randomly allocated to the CWI or control (CON) group. The CWI group underwent a 3-week repeated CWI intervention (12min at 7°C, 4x/week). The CON group did not receive any cold exposure or therapy. Total leukocyte numbers and proportions (neutrophils, basophils, eosinophils, monocytes, lymphocytes) and cardiovascular factors (MAP, HR) were assessed at baseline and after the 3-week intervention period.

**Results:** Total leukocyte count decreased in CWI (*p* = 0.027, 95% CI −2.35 to −0.20 × 10^3^/µL) and CON (*p* = 0.043, 95% CI −2.75 to −0.50 × 10^3^/µL). CWI showed a decrease in neutrophil number (*p* = 0.028, 95% CI −1.55 to −0.25 × 10^3^/µL) and proportion (*p* = 0.046, 95% CI −6.42 to 0.56%). In contrast, CON showed no significant change (*p* > 0.05). No differences were found for other leukocyte subtypes in CWI or CON (all *p* > 0.05). MAP (*p* = 0.028, 95% CI −17 to −8 mmHg) and HR (*p* = 0.027, 95% CI −7 to −2 bpm) were reduced in CWI, whereas CON showed no change (*p* > 0.05).

**Conclusion:** The results suggest no relevant effects of 3-week repeated CWI on leukocyte counts in healthy men. Due to methodological limitations, the effects on the investigated cardiovascular factors remain unclear. Further studies with larger sample sizes are needed to examine the effects on immune function and cardiovascular health.

## 1 Introduction

Cold water immersion (CWI) in the form of cold/ice bathing and cold water swimming has become increasingly popular due to its potential health benefits (Tipton et al., 2017; Manolis et al., 2019; Knechtle et al., 2020; Espeland et al., 2022). In non-cold-adapted humans, initial whole-body CWI at water temperatures <15°C has a stressful physiological impact, potentially leading to a so-called ‘cold shock response’. This acute thermoregulatory reaction to cold exposure is manifested by hyperventilation, cutaneous vasoconstriction, increased muscle shivering, metabolic heat production, and the release of catecholamine and glucocorticoid hormones (Tipton, 1989; Castellani and Young, 2016; Eimonte et al., 2021a). Repeated CWI leads to adaptive processes that attenuate the initial physiological responses (Keatinge and Evans, 1961; Golden and Tipton, 1988; Castellani and Young, 2016). It has been proposed that short-term stress activates the cardiovascular, musculoskeletal, and neuroendocrine systems for fight-or-flight and, under certain circumstances, prepares the immune system for challenges (Dhabhar, 2009; Dhabhar, 2014). Building on this idea, frequent activation of short-term stress through repeated CWI may induce adaptive psychophysiological mechanisms that enhance immune protection, improve cardiovascular health and, in turn, prevent the occurrence of a wide variety of diseases or attenuate the adverse effects of surgical stress (Harper, 2012; Tipton et al., 2017; Manolis et al., 2019; Knechtle et al., 2020; Espeland et al., 2022).

An essential component of the immune system is the rapid recruitment of immune cells to specific sites of infection, wound, surgery or vaccination. The absolute numbers of circulating leukocytes and leukocyte proportions (i.e., percentage of each leukocyte subtype) provide information about the state of leukocyte distribution in the body and activation state of the immune system. Previous investigations indicate that acute short-term stress stimulates the leukocytes to exit the spleen, lung and marginated pool, as well as other organs, to enter the bloodstream (Dhabhar et al., 1995; Benschop et al., 1996; Dhabhar and McEwen, 1997; Dhabhar, 2009; Dhabhar et al., 2012). It has been consistently shown that short-term stress reactions triggered by a single CWI can rapidly alter the numbers and proportions of leukocytes (Janský et al., 1996a; Brazaitis et al., 2014; Eimonte et al., 2021a; Eimonte et al., 2021b). This modification seems to be dose-dependent (Eimonte et al., 2021a): While more extended CWI protocols (60min at 14°C (Janský et al., 1996a) or intermittent cooling with 120min maximum total immersion time at 13–14°C (Brazaitis et al., 2014; Eimonte et al., 2021b)) tended to increase the total leukocyte count, a shorter (10min at 14°C) CWI protocol produced no change (Eimonte et al., 2021a). In addition, studies reported an increase in the proportion of neutrophils (Brazaitis et al., 2014; Eimonte et al., 2021a; Eimonte et al., 2021b), a decrease in the proportion of lymphocytes (Brazaitis et al., 2014; Eimonte et al., 2021a; Eimonte et al., 2021b) and a reduced proportion of monocytes (Brazaitis et al., 2014; Eimonte et al., 2021b). Taken that the leukocyte counts have been demonstrated to fully recover to pre-exposure levels within 6–12 h after CWI, the observed changes are likely to reflect a re-distribution of leukocytes within different body compartments rather than a formation or destruction of these cells (Cox and Ford, 1982; Eimonte et al., 2021a; Eimonte et al., 2021b).

This raises the question of whether frequent CWI, repeated over several weeks, modifies the mobilisation and recruitment of specific leukocytes to enhance immune protection. To our knowledge, only one study has examined the effects of repeated CWI (60min at 14°C, 6-week intervention, 3x/week) on leukocyte counts. This study found a small increase in the monocyte numbers and the proportion of certain lymphocyte subpopulations, but no change in the total leukocyte count (Janský et al., 1996a). As this initial evidence is based on a within-subject design without a control group, the effects of repeated CWI on leukocyte counts are inconclusive.

Habitual winter-swimmers have been shown to have increased numbers of monocytes compared to inexperienced individuals. Since the winter-swimmers often practise ice-cold water swimming in combination with systemic heat (i.e., hot sauna), the observed differences in leukocyte levels suggest adaptive mechanisms in response to thermal stress in general (Dugué and Leppänen, 2000). Therefore, the effects of cold exposure through repeated CWI remain to be fully elucidated. In addition, several studies have pointed out the difficulty of separating the effects of swimming and/or exercise before, during or after CWI. Acute exercise and cold exposure are both complex physiological conditions that stimulate a stress response, and their combined effects may exceed the individual effects of either stimulus (Tipton et al., 2017; Eimonte et al., 2021a; Espeland et al., 2022). Accordingly, it is essential to explore the effects of a static repeated CWI intervention alone to avoid the confounding effect of exercise.

Among other cardiovascular parameters, elevated resting blood pressure and heart rate (HR) are recognised risk factors for the development of cardiovascular disease (Böhm et al., 2015; Liu et al., 2021). In particular, mean arterial pressure (MAP) predicts cardiovascular health and disease in young men (Sesso et al., 2000). Physiological responses during CWI, including blood pressure and HR, are well documented (Tipton, 1989; Janský et al., 1996b). In addition, cold adaptation through repeated CWI has been shown to alter blood pressure responses during CWI (Muza et al., 1988; Janský et al., 1996b). However, the overall effects of repeated CWI and cold adaptation on cardiovascular health are not clarified. Previous research indicates a beneficial effect of cold adaptation on lipoprotein parameters and antioxidative markers (Kralova Lesna et al., 2015). Nonetheless, investigations focusing on the effects of repeated CWI on resting MAP and HR remain to be completed.

Since research has mainly focused on physiological responses to initial CWI, further investigation is needed to examine the effects of repeated CWI on leukocyte counts and cardiovascular factors. Therefore, the aim of this study was to explore the effects of a 3-week repeated CWI intervention on total leukocyte numbers and proportions (neutrophils, basophils, eosinophils, monocytes, lymphocytes) and cardiovascular factors (MAP, HR) in healthy men. Based on previous studies (Janský et al., 1996a; Dugué and Leppänen, 2000), it was hypothesized that a 3-week repeated CWI intervention may increase the number of monocytes. Furthermore, it was hypothesized that a 3-week repeated CWI intervention could potentially reduce resting MAP and HR.

## 2 Methods

### 2.1 Participants

A total of n = 12 men (age: 25.2 ± 4.0 years; height: 177.8 ± 5.6 cm; weight: 73.8 ± 6.5 kg, estimated lower body fat percentage: 16.4 ± 3.5%) volunteered for this study. Participants were included if they were between the age of 18 and 60 years and engaged in recreational activity for 30–60 min, 2–3x/week. Furthermore, they were included if they were non-habituated to CWI (no prior experience with regular systemic or local cold exposure was reported). Participants were excluded if they were smokers, had a cold allergy, had a history of cardiovascular or respiratory disease, had any pain symptoms, or were taking medication. All participants were fully informed of the experimental protocol, the aims, risks and discomforts related to this study, before signing an informed consent form. The study was approved by the Swiss Ethical Committee of Zurich (Req-2021-00989) in accordance with the Declaration of Helsinki (ICH-GCP). The study was conducted during the winter season in the laboratory of the University of Applied Sciences and Arts of Southern Switzerland (RESlab, Landquart, Switzerland).

### 2.2 Study design

The study was based on a randomised controlled design. The participants were randomly assigned by drawing lots to the CWI group (*n* = 6) or the CON (*n* = 6) group. Participant’s characteristics for each intervention group are displayed in Supplementary Table S1. Age, height, mass and estimated lower body fat percentage did not differ significantly between the intervention groups at baseline (BL) (all *p* > 0.05).

### 2.3 Experimental protocol

To our knowledge, there is currently no standardised repeated static CWI intervention protocol specifically designed for healthy individuals to induce meaningful physiological cold adaptation effects. In the CWI literature, cold water is generally defined as a temperature <15°C (Tipton et al., 1991). Post-exercise CWI protocols have demonstrated significant metabolic effects in non-cold habituated individuals when using temperatures between 5-13°C for durations of 10–24 min (Leeder et al., 2012; Hohenauer et al., 2015). Considering these findings, the water temperature and CWI duration were selected within this range. The CWI group underwent a 3-week repeated CWI intervention with CWI sessions 4x/week, on consecutive days. During the CWI, participants sat in a pool (168 cm × 168 cm) filled with cold water (12min at 7 ± 0.5°C) and submerged to the level of the sternum with their arms on the outside. Participants wore swimming trunks. The water temperature was constantly monitored with a multimeter device (Voltacraft MT52, Wollerau, Switzerland). Crushed ice was added if needed. After the immersion, the participants were patted dry and laid in a supine position. The CON group was not allowed to perform any cold exposure or therapy. All participants were instructed to maintain their regular daily habits during the 3-week intervention period, including their usual food intake and caffeine consumption. BL measurements were performed on the first measurement day of the 3-week intervention period, and follow-up (F/U) measurements were taken 2–3 days post-intervention. BL and F/U measurements were performed in the morning. Anthropometric characteristics at BL included height (GPM Stadiometer, Zurich, Switzerland), body mass, and lower body fat percentage estimation. Body mass and lower body fat percentage estimation were measured using a TANITA-TBF 611 scale (Tokyo, Japan). Estimate of lower body fat percentage was chosen because the participants were submerged without arms to sternum level, which mainly affects the lower body. Blood samples were collected, and cardiovascular factors were analysed at BL and F/U.

### 2.4 Leukocyte counts

Venous blood samples were collected from a cubital vein (K2E EDTA, 4000 µL, BD Vacutainer, Plymouth, UK). Samples were swirled, stored at room temperature and analysed for differential blood cell count using flowcytometry (Sysmex, Norderstedt, Germany) within 12 h after collection by the laboratory Dr. Risch (Labormedizinische Analytik FAMH, Buchs, Switzerland). The proportions of each leukocyte subtype (neutrophils, basophils, eosinophils, monocytes, lymphocytes) were calculated as a percentage of the total leukocyte count. The reference values for the hematologic parameters used were taken from a meta-analysis based on practice of a group of large hospitals and a literature review (Herklotz et al., 2006), which are used by the laboratory Dr. Risch (total leukocyte count: 3.9-10.2 10^3^/µL; neutrophils: 1.5-7.7 10^3^/µL, basophils: 0.0-0.2 10^3^/µL; eosinophils: 0.02-0.5 10^3^/µL; monocytes: 0.1-0.9 10^3^/µL, lymphocytes: 1.1-4.5 10^3^/µL, neutrophil proportion: 42–77%, eosinophil proportion: 2–4%, basophil proportion: <2.0%, monocyte proportion: 2.0–9.5%, lymphocyte proportion: 20–44%).

### 2.5 Cardiovascular factors

Resting MAP was assessed after 10min rest in the supine position using an automatic sphygmomanometer monitor (Beurer, BM77, Beurer GmbH, Ulm, Germany) from the left brachial artery. MAP was calculated according to the following formula: MAP = DBP+([SBP-DBP]/3), where SBP is systolic blood pressure and DBP is diastolic blood pressure (Grillo et al., 2021). HR was measured using a Polar watch (Polar, V800, Kempele, Finland) and a Bluetooth chest belt (Polar, H10, Kempele, Finland) at a time interval of 10min and averaged. The Polar V800 is a valid instrument for measuring RR intervals at rest (Giles et al., 2016). MAP and HR were assessed by the same investigator who was not blinded to assignment to the intervention groups. Reference values for resting MAP (70–100 mmHg) were approximated from the normal blood pressure (SBP: <120 mmHg SBP, and DBP: <80 mmHg) as recognized by the American Heart Association (Whelton et al., 2018). A mean reduction of minimally 5–10 mmHg for SBP, or 3–5 mmHg for DBP can be considered a clinically meaningful reduction in clinical trials (Kandzari et al., 2022). Accordingly, this study’s potential reductions of MAP of this magnitude were considered meaningful. Resting HR was considered normal between 50–80 bpm, as approximated from reports of resting HR in healthy men (Ostchega et al., 2011; Quer et al., 2020).

### 2.6 Statistical analysis

Statistical analysis was performed using IBM SPSS Statistics (29, IBM Corp). Demographic data were reported descriptively (mean ± SD). Independent t-tests were used to observe between-group differences at BL. Given the small sample size (*n* = 12), a non-parametric Wilcoxon matched-pairs signed rank test was used to compare the before-and-after differences for each variable. Effect sizes (Cohen’s d) were calculated using the formula $r=\frac{\left|Z\right|}{\sqrt{n}}$ (Rosenthal, 1984) and defined as follows: small: ≤0.2; medium: ≤5; large: ≤0.6 (Cohen, 1998; Fritz et al., 2011). In all analyses, statistical significance was set at *p* < 0.05. Figures were created using Prism (9, Graphpad, Software Inc.). Post hoc power analysis for Wilcoxon signed-rank test (matched pairs, 2-tailed, normal parent distribution, *α* = 0.05, *n* = 12) was performed for each outcome using G*Power (version 3.1.9.6, Franz Faul, Germany).

## 3 Results

### 3.1 Leukocyte counts

**FIGURE 1:**
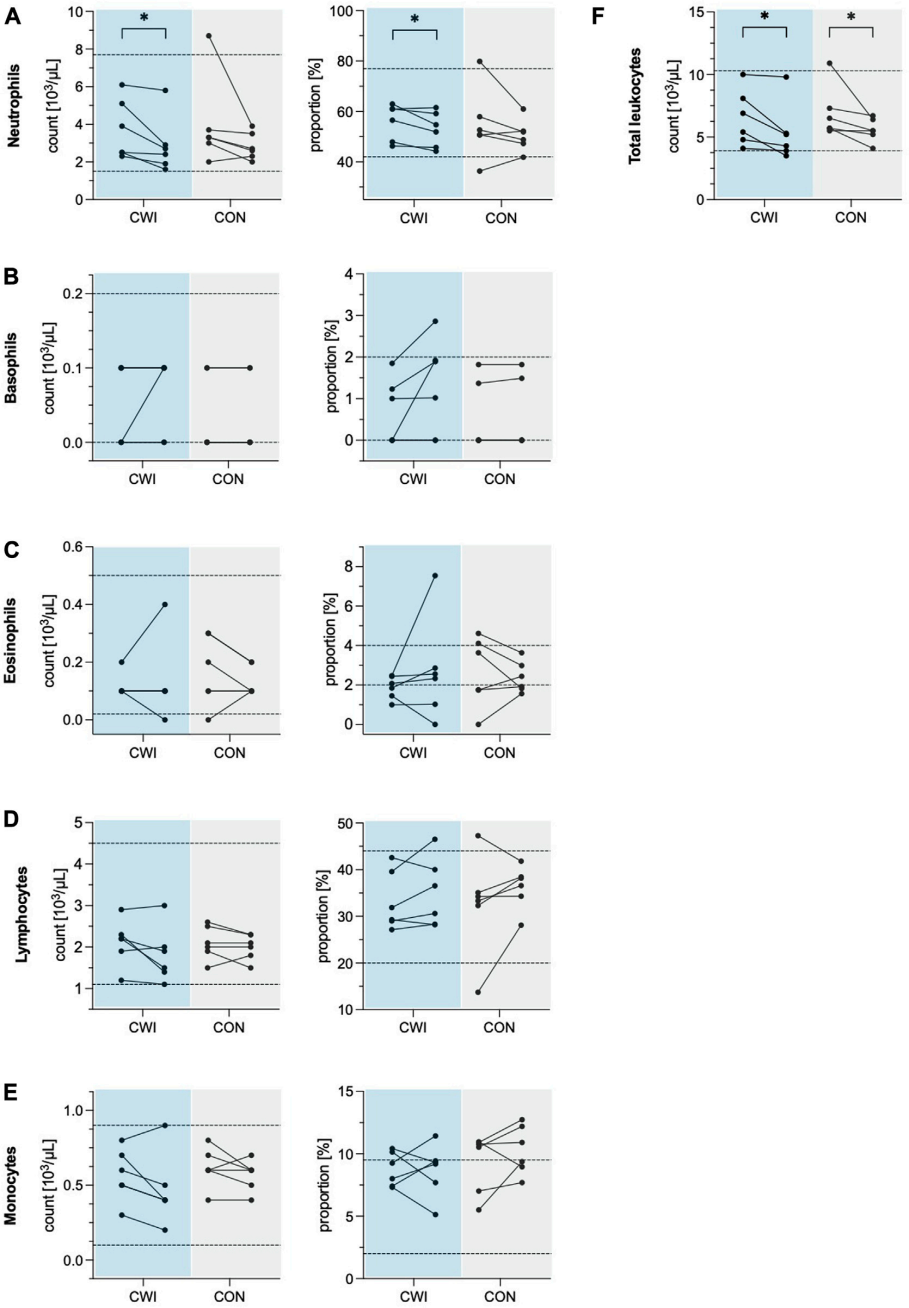
Leukocyte numbers and proportions of the cold water immersion (CWI) and control group (CON), at baseline (BL, left) and after the intervention (follow-up, F/U, right). Solid lines connect individual participants’ values. Dashed lines represent the upper and lower limits of the reference values for adults (18–65 years). **(A)** Neutrophils. **(B)** Basophils. **(C)** Eosinophils. **(D)** Lymphocytes. **(E)** Monocytes. **(F)** Total leukocytes. *p < .05, **p < .01, ***p < .001. N = 12.

Total leukocyte count decreased significantly in CWI (*p* = 0.027, r = 0.637,1-ß = 0.49) with a median difference of −1.10 × 10^3^/µL (95%CI −2.35 to −0.20), and CON (*p* = 0.043, r = 0.584, 1-ß = 0.43) with a median difference of −0.8 × 10^3^/µL (95%CI −2.75 to −0.50). Leukocyte differential count revealed a significant decrease in the number of neutrophils in CWI (*p* = 0.028, r = 0.635, 1-ß = 0.50) with a median difference of −0.65 × 10^3^/µL (95%CI −1.55 to −0.25). At the same time, no change was observed in CON (*p* = 0.075, r = 0.514, 1-ß = 0.35). The neutrophil proportion was reduced significantly in CWI (*p* = 0.046, r = 0.575, 1-ß = 0.42) with a median difference of −2.77% (95%CI −6.42 to 0.56). In contrast, CON did not show a significant change (*p* = 0.249, r = 0.332, 1-ß = 0.18). There were no significant differences in the numbers or proportions of basophils, eosinophils, monocytes or lymphocytes in CWI or CON (all *p* > 0.05). Complete results of the leukocyte counts are displayed in Table 1. Participants’ values are shown in Figure 1.

**TABLE 1:** Leukocyte counts at baseline (BL) and after the intervention period (follow-up, F/U). Wilcoxon matched-pairs signed rank test results indicate the before-after differences for each intervention group.

	Unit	Group	Median		Median difference (F/U-BL)	BCa 95%CI		Z-value	*p*-value	Effect size
			BL	F/U		Lower	Upper			
Total leukocytes	count [10^3^/µL]	CWI	6.15	4.75	−1.10	−2.35	−0.20	−2.207	0.027*	0.637
		CON	6.10	5.50	−0.80	−2.75	−0.50	−2.023	0.043*	0.584
Neutrophils	count [10^3^/µL]	CWI	3.20	2.55	−0.65	−1.55	−0.25	−2.201	0.028*	0.635
		CON	3.30	2.65	−0.65	−2.90	0.05	−1.782	0.075	0.514
	proportion [%]	CWI	58.75	53.32	−2.77	−6.42	0.56	−1.992	0.046*	0.575
		CON	51.70	50.35	−3.67	−12.43	3.50	−1.153	0.249	0.332
Basophils	count [10^3^/µL]	CWI	0.05	0.10	0.00	-	-	−1.000	0.317	0.289
		CON	0.00	0.00	0.00	-	-	0.000	1.000	0.000
	proportion [%]	CWI	0.50	1.45	0.34	0.00	1.38	−1.826	0.068	0.527
		CON	0.00	0.00	0.00	-	-	−1.000	0.317	0.289
Eosinophils	count [10^3^/µL]	CWI	0.10	0.10	0.00	-	-	−0.447	0.655	0.129
		CON	0.15	0.10	−0.05	−0.10	0.00	−1.000	0.317	0.289
	proportion [%]	CWI	1.97	2.44	0.18	−0.71	3.04	−1.153	0.249	0.333
		CON	2.70	2.18	−0.41	−1.40	0.68	−0.524	0.600	0.151
Lymphocytes	count [10^3^/µL]	CWI	2.20	1.70	−0.20	−0.70	0.10	−1.378	0.168	0.398
		CON	2.05	2.05	−0.10	−0.25	0.00	−0.921	0.357	0.266
	proportion [%]	CWI	30.58	33.58	1.38	−1.06	4.65	−1.153	0.249	0.334
		CON	33.79	37.38	3.31	0.08	5.87	−1.363	0.173	0.393
Monocytes	count [10^3^/µL]	CWI	0.55	0.40	−0.10	-	-	−1.667	0.096	0.481
		CON	0.60	0.60	−0.05	−0.10	0.00	−1.134	0.257	0.327
	proportion [%]	CWI	8.63	9.24	0.03	−2.19	2.03	−0.314	0.753	0.09
		CON	10.65	10.14	1.17	0.14	1.82	−1.153	0.249	0.333

BL = baseline, F/U = follow-up, BCa 95% CI = Bias-corrected and accelerated 95% confidence interval, CWI = cold water immersion, CON = control, *p < .05, **p < .01, ***p < .001 (asymp. sign. 2-tailed).

### 3.2 Cardiovascular factors

**FIGURE 2:**
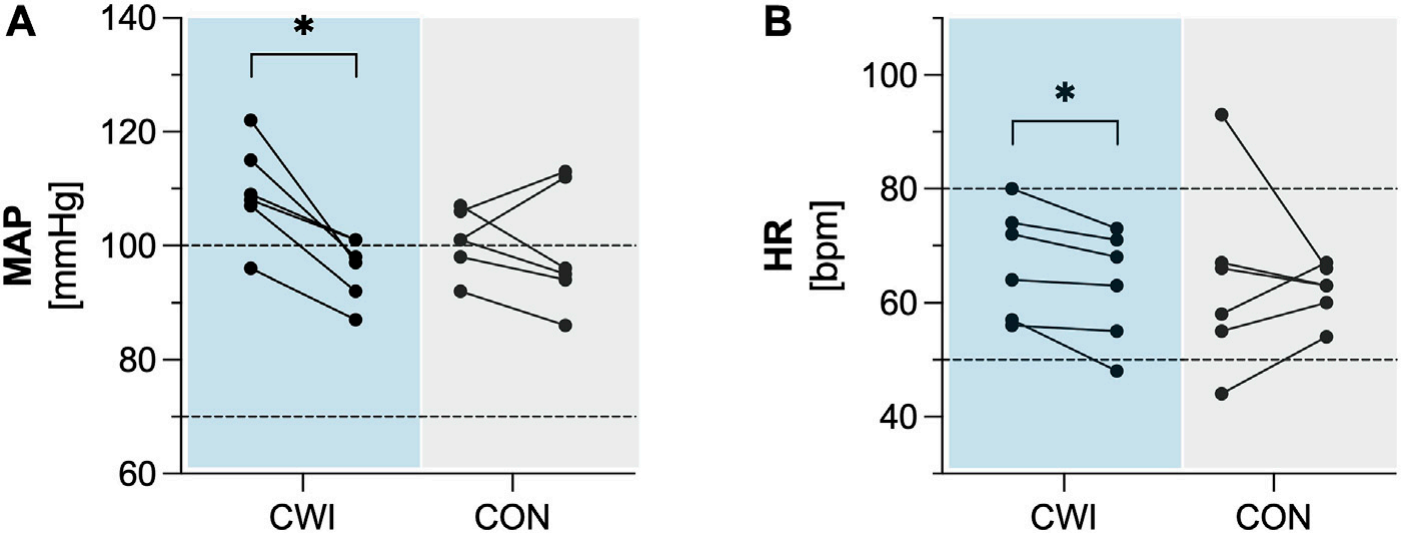
Cardiovascular factors **(A)** Mean arterial pressure (MAP). **(B)** Heart rate (HR) of the cold water immersion (CWI) and control group (CON) at baseline (BL, left) and after the intervention (follow-up, F/U, right). Solid lines connect individual participants’ values. Dashed lines represent the upper and lower limits of reference values for adults. *p < .05, **p < .01, ***p < .001. N = 12.

CWI showed a significant decrease in MAP (*p* = 0.028, r = 0.635, 1-ß = 0.50) with a median difference of −12 mmHg (95%CI −17 to −8), as well as in HR (*p* = 0.027, r = 0.637, 1-ß = 0.50) with a median difference of −4 bpm (95%CI −7 to −2). In contrast, CON did not show a significant change in MAP (*p* = 0.833, r = 0.061, 1-ß = 0.06) or HR (*p* = 0.753, r = 0.091, 1-ß = 0.06)). Complete results of the cardiovascular factors are available in Table 2. Participants’ values are shown in Figure 2.

**TABLE 2:** Cardiovascular factors at baseline (BL) and after the intervention period (follow-up, F/U). Wilcoxon matched-pairs signed rank test results indicate the before-after differences for each intervention group.

	Group	Median		Median difference (F/U-BL)	BCa 95%CI		Z-value	*p*-value	Effect size
		BL	F/U		Lower	Upper			
MAP [mmHg]	CWI	109	98	−12	−17	−8	−2.201	0.028*	0.635
	CON	101	96	−5	−9	9	−0.211	0.833	0.061
HR [bpm]	CWI	68	66	−4	−7	−2	−2.207	0.027*	0.637
	CON	62	63	1	−16	10	−0.314	0.753	0.091

BL = baseline, F/U = follow-up, BCa 95% CI = Bias-corrected and accelerated 95% confidence interval, CWI = cold water immersion, CON = control, *p < .05, **p < .01, ***p < .001 (asymp. sign. 2-tailed).

## 4 Discussion

The aim of this study was to investigate the effects of repeated CWI on leukocyte counts and cardiovascular factors. Blood samples from healthy men were analysed, and cardiovascular factors were assessed at BL and after a 3-week repeated CWI intervention compared to a CON group. The main findings of this exploratory study suggest that a 3-week repeated CWI intervention has no relevant effects on leukocyte counts.

The current study revealed a minimal reduction in total leukocyte counts in both the CWI and CON groups, suggesting that repeated CWI did not result in relevant changes in the total number of circulating leukocytes. This finding is consistent with previous research by Janský et al. who also showed no changes in the total leukocyte count after a 6-week repeated CWI intervention (Janský et al., 1996a). In the same study, Janský et al. reported a slight increase in monocyte numbers (Janský et al., 1996a) and Dugué et al. found that winter-swimmers exposed to thermal stress had higher numbers of monocytes compared to non-habituated individuals (Dugué and Leppänen, 2000). Contrary to these observations, a 3-week repeated CWI intervention in this present study had no significant effect on monocyte numbers but only resulted in a minimal decrease in neutrophil numbers (median difference: −0.65 × 10^3^/µL) and proportion (median difference: −2.77%). Considering that a mean peripheral neutrophil number of 4.4 (4.3–4.9) 10^3^/µL is considered normal for healthy adults (Zini et al., 2011) and taken that neutrophil counts show dynamic fluctuations (Scheiermann et al., 2015), the observed reductions in neutrophils in CWI do not seem relevant.

Research has predominantly focused on the immediate physiological responses to a single CWI (Brazaitis et al., 2014; Eimonte et al., 2021a; Eimonte et al., 2021b). It has been consistently shown that a single CWI triggers short-term adaptive physiological mechanisms involving the re-distribution of immune cells rather than the formation or destruction of these cells (Cox and Ford, 1982; Eimonte et al., 2021a; Eimonte et al., 2021b). According to Dhabhar et al. (2014), short-term stress is defined as lasting minutes to hours, and chronic stress is persistent for hours each day for weeks or months (Dhabhar and McEwen, 1997; Dhabhar, 2014). Short-term stress can stimulate adaptive physiological responses that enhance immune protection by modulating the innate/primary and adaptive/secondary immune responses, including changes in immune cell trafficking, maturation and function. However, long-term or chronic stress is considered harmful because it suppresses or dysregulates the innate and adaptive immune responses (Dhabhar, 2014). Based on this concept, it has been proposed that regular activation of the short-term stress response by repeated CWI could mediate adaptive physiological mechanisms that enhance immune protection (Harper, 2012; Tipton et al., 2017; Manolis et al., 2019; Knechtle et al., 2020; Espeland et al., 2022). In the present study, it was assumed that short-term re-distribution of immune cells occurred 6–12 h after each CWI, as it was shown previously (Eimonte et al., 2021a; Eimonte et al., 2021b). However, since there were no relevant changes following the 3-week intervention period, repeated CWI does not appear to alter the numbers or proportions of leukocytes.

A critical issue to consider is whether the repeated CWI protocol used in this study was adequate to stimulate potential changes in leukocyte counts. In the study of Janský et al., it was concluded that the cold stimulus (60min at 14°C, 6-week intervention, 3x/week) may not have been strong enough to induce significant changes (Janský et al., 1996a). However, there is conflicting evidence to support this interpretation. It is reasonable to assume that the 3-week repeated CWI protocol used in the present study (12min at 7°C, 4x/week) was appropriate for two reasons: 1) Given that the participants were non-cold adapted, the frequency and the intensity of the cold stimulus were relatively high; 2) The participants experienced several minutes of sustained shivering after CWI. Therefore, it could even be speculated whether repeated CWI protocols could potentially induce chronic stress as defined above (Dhabhar and McEwen, 1997; Dhabhar, 2014). Nonetheless, responses to CWI vary widely between individuals, and an optimal dose-response relationship for non-cold-adapted humans has not yet been defined (Tipton et al., 2017). Although changes in mature leukocyte mobilisation may occur within a few days (Scheiermann et al., 2015), meaningful adaptations in leukocyte mobilisation and recruitment associated with repeated CWI may take longer than 3 weeks.

To our knowledge, this is the first study to explore the effects of repeated CWI on resting MAP and HR. The results of this study showed a significant reduction in MAP and HR for CWI but not for CON. Even though the reduction of MAP (median difference: −12 mmHg) found in CWI might be considered clinically meaningful (Kandzari et al., 2022), this finding must be interpreted with caution given the following considerations. Resting MAP was generally elevated in all participants, with the BL measurements being exceptionally high in the CWI group (median: 109 mmHg). Participants in the CWI group were measured on the first day of the intervention period, presumably contributing to the elevated blood pressure due to nervousness associated with their first CWI session. As the CWI group became more familiar and accustomed to the experimental protocol, their blood pressure may have decreased. Therefore, this reduction cannot be directly related to the repeated CWI. Moreover, in this study blood pressure was only measured once for each time point. According to blood pressure measurement guidelines for adults, resting blood pressure levels should be estimated based on the mean of ≥2 assessments on ≥2 occasions (Whelton et al., 2018). Consequently, due to the methodological limitations in this exploratory study, the effects of repeated CWI on resting MAP and HR remain inconclusive and require verification using adjusted measurement protocols.

The observations in this study suggest no relevant effect of repeated CWI on leukocyte counts. Due to the preliminary and exploratory nature of this study, the conclusions are provided considering several limitations. 1) A *post hoc* power analysis revealed that the study was underpowered to reliably detect the effects of interest. It is therefore essential to validate the findings with a larger sample size. 2) Since this study focused solely on males, the absence of females in this sample may restrict the generalisability of the findings. Future research should examine the effects in both males and females to provide a more comprehensive understanding of the physiological responses to repeated CWI. 3) Participants in the CON group were free-living, making it impossible to separate the physiological effects of the hydrostatic pressure exerted from the water itself (Wilcock et al., 2006). Therefore, future research could consider using a thermoneutral bath to test the specific contribution of exposure to cold water. 4) Even though leukocyte counts provide a valuable insight into immune system function, their clinical importance in the context of CWI is not apparent (Tipton et al., 2017; Knechtle et al., 2020). The numbers and proportions of circulating leukocytes vary and reflect the current need for specific leukocyte subtypes at a given time point (Scheiermann et al., 2015). Consequently, leukocyte counts do not provide a complete picture of the immune function and should not be used as a sole marker in future studies.

To better understand cold adaptation responses and potential immune stimulation by repeated CWI over time, it is recommended that future investigations include measurements of acute physiological responses after each CWI session. This would provide insight into the dynamic changes in leukocyte counts (Scheiermann et al., 2015). There is evidence that cold-adapted humans may be more resistant to certain illnesses and infections (Kormanovski et al., 2010; Collier et al., 2021). Although this information is based on participants’ self-reports of illness, infection or general wellbeing, such additional information could be included in similar studies.

Various CWI protocols have been used over the past decades. The magnitude of physiological responses to CWI depends on the protocol (i.e., water temperature, duration, time points of measurements) and participant characteristics (i.e., age, body fat percentage, degree of cold acclimation, training status, health style, social interaction, mindset) (Tipton et al., 2017; Espeland et al., 2022). In order to investigate physiological responses to CWI more reliably, it is necessary to establish standardised CWI protocols and to account for confounding variables. Finally, this study used a static CWI protocol to avoid the confounding effects of exercise. Nevertheless, investigating the synergistic effects of CWI and exercise, particularly in the context of cold water swimming, remains an important research area (Harper, 2012; Tipton et al., 2017; Manolis et al., 2019; Knechtle et al., 2020; Espeland et al., 2022).

## Data Availability

The original contributions presented in the study are included in the article/Supplementary Material, further inquiries can be directed to the corresponding author.
