# Supplementary material for: Effects of 3-week repeated cold water immersion on leukocyte counts and cardiovascular factors: an exploratory study
Source: Front Physiol. 2023 Aug 29;14:1197585. doi: 10.3389/fphys.2023.1197585 (PMC10497764; doi:10.3389/fphys.2023.1197585)
Supplement: Supplementary file 1 [file Table1.docx]

Supplementary Material

Effects of 3-week repeated cold water immersion on leukocyte counts and cardiovascular factors: An exploratory study

Ninja Versteeg^1*^, Ron Clijsen^1,2,3,4^, Erich Hohenauer^1,2,3,4,5^

*** Correspondence:** Ninja Versteeg, ninja.versteeg@supsi.ch

# Supplementary Figures and Tables

**Supplementary Table 1.** Summary of the participant's characteristics of the intervention groups

|  | CWI (n=6) | CON (n=6) | Independent t-test between groups (p-value) |
| --- | --- | --- | --- |
| Age [years] | 26.3±3.7 | 24.0±4.3 | >0.05 |
| Height [cm] | 178.8±7.5 | 176.7±3.2 | >0.05 |
| Mass [kg] | 74.9±8.0 | 72.6±5.2 | >0.05 |
| Lower body fat [%] | 16.8±2.6 | 16.0±4.6 | >0.05 |
| Data presented as mean±SD, CWI = cold water immersion, CON = control | | | |
